# How parents derive their informed role preferences for end-of-life medical decision-making for (unborn) children in perinatology: a meta-ethnography of qualitative interview studies

**DOI:** 10.1186/s12910-026-01605-1

**Published:** 2026-09-18

**Authors:** Katja Kuehlmeyer, Maria F. Beyer, Pezi Mang, Andreas W. Flemmer, Esther S. Schouten

**Affiliations:** 1 Institute of Ethics, History and Theory of Medicine, LMU Munich, Lessingstraße 2, 80336 Munich, Germany; 2 Division Neonatology, Dr. von Hauner Children’s Hospital, LMU University Hospital Munich, Marchioninistrasse 15, 81377 Munich, Germany

**Keywords:** Neonatology [MeSH], Parents [MeSH], Palliative care [MeSH], Decision-making, Shared [MeSH], Abortion, Induced [MeSH], End-of-life issues, Termination of pregnancy, Doctor-patient communication, Ethics

## Abstract

**Background:**

End-of-life medical decision-making (EOL MDM) for (unborn) children is an integral part of perinatology. Medico-ethical guidelines for EOL MDM increasingly recommend shared decision-making (SDM) with parents. One argument for SDM is that parents prefer it. However, when SDM is distinguished from other forms of collaborative EOL MDM (cEOL MDM), parents’ role preferences are not uniform. We aimed at exploring 1) how parents who have experience with cEOL MDM for their (unborn) child derive their preference for a certain role and 2) which role they prefer.

**Methods:**

For this purpose, we used Meta-Ethnography (ME) to synthetize qualitative studies on parents’ perspectives on EOL DM.

**Results:**

The synthesis of 16 articles resulted in a line of argument which explains a process of preference-formation: Parents who experience a certain role allocation can evaluate it differently at different time points, and may express their experience-based, informed preferences towards future decision-making (DM) as a willingness (not) to repeat their experience. At least four different practice models can be distinguished based on parents’ experiences: the tacit approval model, the recommendation acceptance model, the shared deliberation model and the preference determination model. Despite its prevalence in the theoretical literature and recommendation in several international guidelines, the shared deliberation model has only been described by parents in two studies. Parental preference-formation can be shaped through a process of dissonance reduction and a process of meaning-making. Most parents express the willingness to repeat their experience, regardless of their role in the cEOL MDM.

**Conclusion:**

As adopted DM models, and consequently the level of parental involvement in perinatal end-of-life decision-making (EOL-MDM), as well as parents' preferences, vary considerably across different contexts, it is questionable whether guidelines should prescribe only one DM model. Rather than aiming to give parents maximum decision-making authority, physicians should adapt their choice of model to the specific situation. While parents’ expectations are one factor in deciding on the appropriate model, other factors, such as the infant's prognosis, also need to be considered.

**Supplementary Information:**

The online version contains supplementary material available at https://doi.org/10.1186/s12910-026-01605-1.

## Background

End-of-life medical decision-making (EOL MDM) for unborn children and neonates is conducted regularly in perinatology [1]. This often includes the decision to terminate a pregnancy or to provide palliative care and to forgo (withhold or withdraw) life-sustaining treatment (LST) for a newborn [2]. Such decisions are usually made when a(n) unborn child has a serious health impairment with an unfavorable prognosis caused by congenital malformations, genetic disorders, prenatally acquired diseases, severe brain injury or preterm birth and an unlikely or a highly uncertain potential to benefit from medical treatment [3, 4].

Perinatal EOL MDM for (unborn) children can pose moral dilemmas for both parents and physicians [5]. In various countries, medico-ethical guidelines here prescribe shared decision-making (SDM) between parents and physicians [3, 6–11]. The call for greater parental involvement in EOL-MDM dates back to a time when parents were largely excluded from this process [2].

SDM with parents is not justified by the norm of respecting parental autonomy, but rather by parental authority in determining the right course of action for their child [4]. Parental authority has its limits, as the child’s current and future well-being (or “best interest”) is of paramount importance in such decisions [4, 12, 13]. Parents may base their considerations not only on health-related values, but also on family values or religious beliefs that can include different opinions about what constitutes a good life [14, 15].

There is a lack of consensus on the degree to which EOL MDM should be shared between physicians and parents. Critical voices argue that parents could feel overburdened by the suggestion to participate in the EOL MDM process and worry about a potential of harm due to the emotional and potentially traumatic nature of the experience or feelings of guilt after deciding to forgo LST [16, 17]. Proponents argue that parental participation in EOL MDM could have a positive influence on the parents’ grieving process [18]. They also point out that parents are particularly affected by such decisions and that they preferred SDM over other decision-making (DM) models [4, 18, 19].

One difficulty in assessing whether parents actually prefer SDM is that there is no uniform understanding of the concept "SDM". The defining feature is the collaboration between parents and physicians with the goal of acknowledging and incorporating the parents’ informed treatment preferences and values in the MDM process [20–22]. Yet, there are several ways or models that show how to act in accordance with that aim, with different moral implications and responsibilities for the parties involved [23, 24].

There have been previous qualitative research syntheses (QRS) that analyzed publications of qualitative studies on parents’ perspectives on EOL MDM in perinatology, yet the results do not include a detailed description of parental preference formation [25–27]. In this meta-ethnography, we aim to provide a comprehensive synthesis of the qualitative interview studies on collaborative EOL MDM (cEOL MDM) in perinatology from the perspective of involved parents.

We specifically aim to answer the research questions: (1) How do parents who have experience with cEOL MDM for their (unborn) child derive their preference for a certain role? (2) Which role do they prefer?

To reduce the potential for confusion, from now on we use the term collaborative EOL MDM (cEOL MDM) to refer to DM that is based on a collaboration between physicians and parents. We use and describe terms that describe a certain model for collaboration.

## Methods

### Approach to meta-synthesis

Based on a comparative review of twelve methods for qualitative research synthesis, we chose meta-ethnography (ME) as the most appropriate approach for qualitative research synthesis in this study [28]. It offers advantages in the development of theories in which different concepts can be integrated into a larger whole. Noblit and Hare (1988) initially developed ME for educational research. It was later adopted by other fields and is now the most commonly used method for QRS in health and social care research [29]. It is suitable for the QRS of studies with different methodological approaches, including qualitative interview studies [30]. ME can be used, in particular, in fields where issues have already been extensively researched, such as cEOL-MDM in perinatology [30]. The method is clearly described, and experts have ensured that the guidelines are accessible [31].

The purpose of this ME was to examine qualitative interview studies involving parents who had experienced EOL MDM for their (unborn) child. The reporting of our ME follows the eMERGe-guideline, which was developed for this method [31].

### Meta-ethnography

ME consists of an iterative process with seven phases that provides the context for comparing the studies [32]. Through a translation process, concepts are elevated to higher levels of abstraction [33]. First-, second- and third-order constructs structure this process: first-order constructs are the literal quotations from the study participants, second-order constructs are the interpretations of the study authors, and third-order constructs are the interpretations of the authors of this article.

### Phases of meta-ethnography

#### Getting started

Our analysis was not based on any previous theory or concept.

Our research team consisted of a social scientist and ethicist (KK) without work experience in the neonatal intensive care unit (NICU), a nurse and (public) health researcher (PM) with work experience in perinatology and three neonatologists with 3, 8 and 35 years research experience and work experience in the NICU (MB, ES, AF).

In the first phase, we (KK, MB, ES) identified the research gap and formulated the research questions that guided all subsequent six steps.

#### Deciding what is relevant

In the second phase, we (MB, ES) conducted a systematic literature search and reviewed potentially relevant peer review publications in the databases Pubmed, PsycINFO, and CINAHL. We searched for published articles from 01.01.1990 until 30.11.2023 and included all studies that met the following inclusion criteria: (1) Qualitative research methodology, (2) interview studies, (3) results regarding parents’ perspectives on their participation in an actual (not a hypothetical) process of EOL MDM between them and members of the medical team, which was realized in conversations, (4) when deciding on the termination of pregnancy or the withholding or withdrawal of LST of (5) (unborn) children before, during or after birth. We only considered articles in German or English. Information about the search string to the example of PubMed is given in Appendix 1. The full electronic search strategy is available on request. We identified the articles that met the inclusion criteria. The quality of the identified articles was further assessed based on the study reports [34]. The quality appraisal was conducted by the three researchers (KK, MB, ES) independently and discussed until consensus was reached. None of the selected articles was excluded after quality appraisal. Yet we identified articles that had the potential to contribute more to our research interest [35–37] than others [38–43] due to their research foci. All included articles were synthesized.

#### Reading included studies

Three authors (KK, MB, ES) read the articles individually several times and discussed the most relevant aspects. We selected studies for joint discussion on a consecutive basis and discussed their differences and commonalities.

#### Determining how studies are related

We (KK, MB; ES) identified the key concepts beginning with the studies that contained the greatest variety. As described by Campbell et al. (2003), we identified an index article - the paper Caeymaex et al. (2011) - for the translation [40, 44]. We selected it as the index article because it presented a thorough and well-developed concept of cEOL MDM. The study was of high quality, with transparent reporting and detailed findings. We compared all articles repeatedly to determine the relationships between the concepts, grouped similar concepts together and later mapped all the relevant concepts.

#### Translating studies into one another

In order to relate the concepts to each other, we (KK, MB, ES) first identified and constructed the second-order concepts — the authors’ interpretations of what the participants meant. Next, we translated the meaning of the concepts from one study into another study, resulting in a conceptual dictionary. The conceptual dictionary was used to bridge the gap between the various interpretations of relevant aspects of EOL MDM across different research contexts. The Results section provides detailed explanations of our understanding of these concepts.

#### Synthetizing translations

The reciprocal translation resulted in third-order constructs. We (KK, MB, ES) then formed a line of argument synthesis.

#### Expressing the synthesis

In the last step, we (KK, MB, PM, AF, ES) elaborated our line of argument synthesis.

## Results

### Sample

**Fig. 1:**
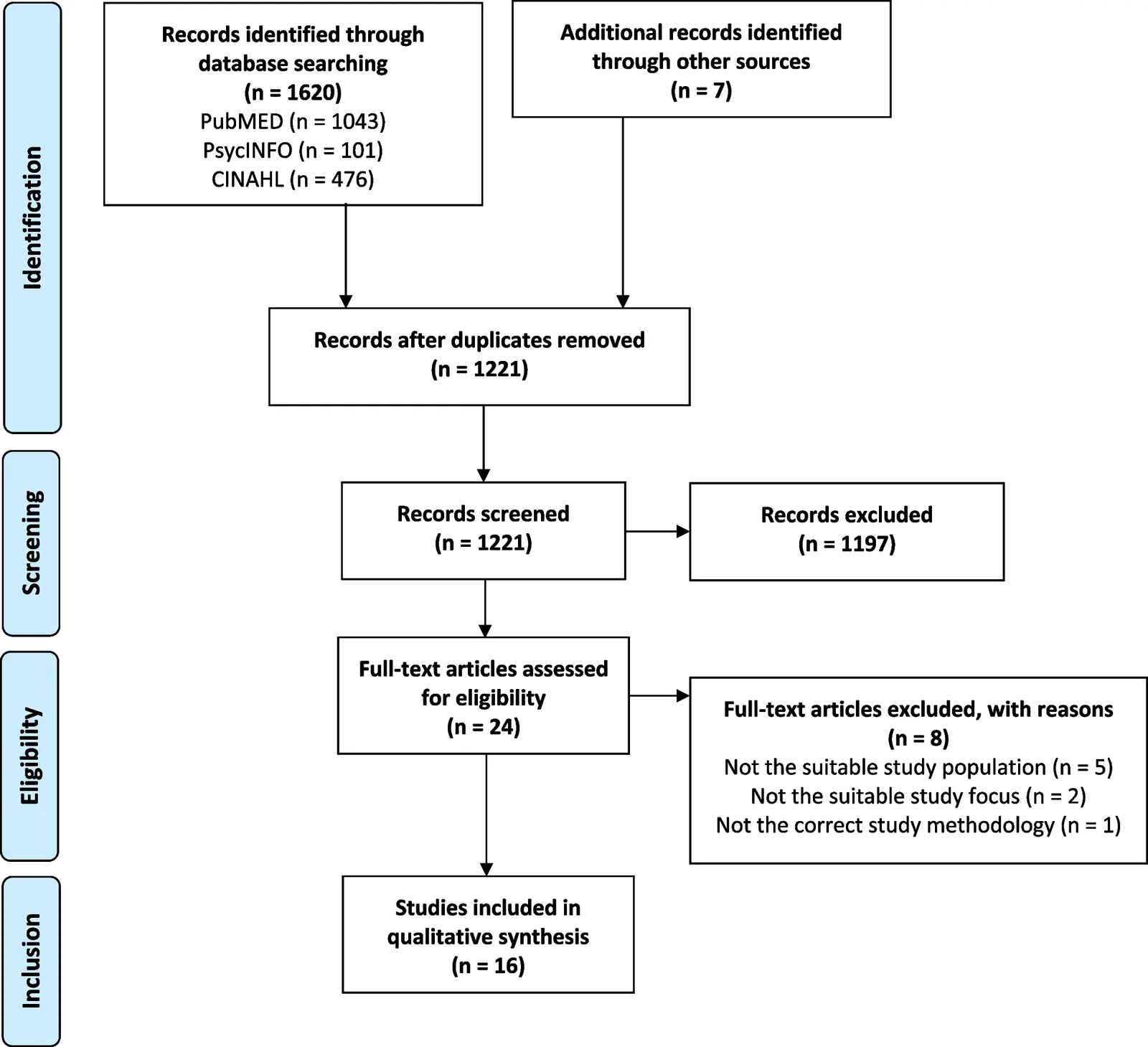
PRISMA flow diagram of the systematic literature search

The PRISMA flow diagram of the systematic literature search is shown in Fig. 1. We included 16 of 1221 screened papers (see Table S1 in Appendix 2 for an overview of the articles' characteristics).

The studies were conducted in either North America (*n* = 11) [35, 38, 39, 41–43, 45–49], Europe (*n* = 4) [23, 36, 40, 50] or both (*n* = 1) [37]. They took place in six different countries (USA, Canada, Norway, France, Switzerland and the Netherlands) and across at least 28 different facilities. Five publications were shared by authors who were supposedly part of the same study or research group and it is unclear whether they are reporting data from the same or from different samples [35, 38, 39, 45, 46]. Participants were either enrolled in the study prospectively, if they were identified as being at risk of preterm birth, or retrospectively, after their child had died in the delivery room or in the NICU.

In total, the studies included at least 408 cases with 515 parents (361 mothers) of 337 infants who had previously been involved in conversations about perinatal EOL MDM either before or/and after the birth of their infant (pre- or/and postnatal decisions). Prenatal decisions concerned options such as termination of pregnancy, pregnancy management, mode of delivery or infant resuscitation [23, 39, 41, 43, 45–47, 49, 50]. Postnatal decisions involved foregoing intensive care including for example artificial respiration or infant resuscitation [23, 36–38, 42, 46, 48].

Some studies had one time point for data collection [23, 35–37, 40, 42, 43, 45, 47, 48, 50], while others had multiple time points [38, 39, 41, 46, 49]. The time intervals between the EOL MDM between parents and physicians and the interviews varied considerably between the individual studies, ranging from few hours [35, 38, 39, 41, 45–47] to eight years [36] after the DM process. Most interviews with parents were conducted face-to-face [23, 35–42, 45–47, 49, 50] and/or over the telephone [40, 42, 43, 48, 49], and were complemented by information from medical charts of the mother and/or the infant [35, 37, 38, 40, 42, 43, 46, 48]. A considerable number of studies also incorporated conversations and interviews with physicians, nurses and other healthcare professionals [23, 35–38, 41, 46, 47].

### Derivation of informed role preferences for cEOL MDM

**Fig. 2:**
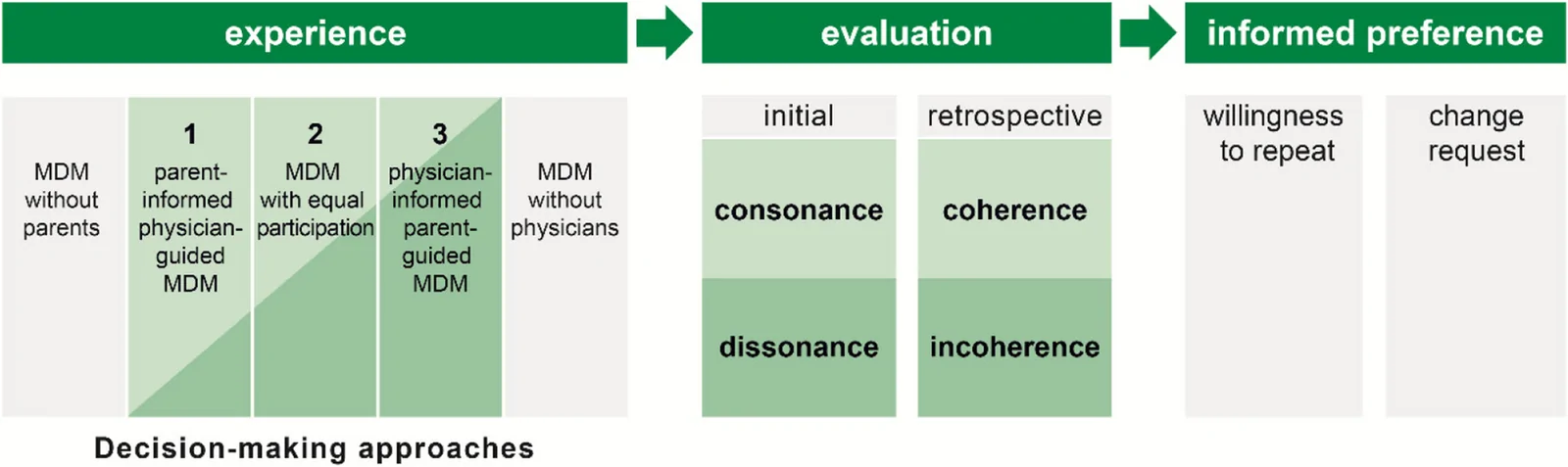
Derivation of a preference for a certain role in cEOL MDM

In this ME we derived an overarching theory from the included articles in our sample. This theory of development of informed preferences of parents for their role in cEOL MDM is visualized in Fig. 2. The figure shows a three-step process from left to right structured by the parts "experience", "evaluation" and "informed preference". In the "experience" block, the two grey areas on the two ends depict non-collaborative MDM. The three areas in the middle depict various forms of collaborative MDM. The dark green ramp illustrates a growing involvement of parents in DM from left to right. The “evaluation” block shows the two time points at which parents can evaluate their experience: initially (immediately after the DM conversation) and retrospectively (after the conclusion of the process). It also shows the results of their evaluation – instantly either consonance or dissonance and retrospectively either coherence or incoherence. In the "informed preference" block, parents’ preferences can entail a willingness to repeat their experience or a request for a change of the applied DM model. We will explain each part in detail in the following sections.

### Experience: The spectrum and areas of cEOL MDM

In order to understand the spectrum of cEOL MDM, it is important to first describe what lies outside of the spectrum. Outside of the spectrum of cEOL MDM, depicted in the grey boxes, there are two forms in which one party acts without informing the other of their (planned) actions. EOL MDM by physicians without parental involvement (in the grey box on the left) could occur, for example, in an emergency situation where a mother is unconscious and the child’s father cannot be reached. EOL MDM by parents without the involvement of a physician (in the box on the right) could for example, when a mother gives birth at home and the decides not to call an emergency service even though the child has health problems. While theoretically possible, such situations occur rarely in practice. Between the two grey boxes lies the spectrum of cEOL MDM.

On the left side on the spectrum, parental involvement in cEOL MDM entails informing parents that a physician will have to discontinue LST either instantly or later, should their child's condition deteriorate. During such a conversation, parents can influence the decision, by expressing their concerns or opinions, which the physicians then can take into account. This option is depicted as area 1 and named *parent-informed physician-guided MDM*.

On the right side, parental involvement means parents make a decision after getting information about the child's condition, treatment options, possible treatment goals and prognosis which is then implemented by physicians and the interprofessional health care team. This option is depicted as area 3 and named *physician-informed parent-guided MDM*. In both cases, one party makes a decision outside of the parent-physician-conversation and seeks the other party’s agreement.

In the middle, parents and physicians participate equally in EOL MDM and SDM authority and responsibility, with equally strong leadership from both parties (area 2, *MDM with equal participation*). Both parties negotiate rules for DM as well as the decision itself. In the second area the extent of each party’s contribution may vary at certain stages of the process. Yet, it is necessary that both parties are concerned with proposing an action plan and reaching an agreement based on open discussions instead of communicating and agreeing on a previously made decision.

The decisive factor in determining the placement of a specific case is the action taken. If both parties first negotiate the rules but then one party acts by deciding alone, the case either falls into the first or the third area. For example, if a mother delegates a decision to a physician, we do not consider it to be parent-guided, but rather physician-guided EOL MDM. In our terminology, this would be parent-informed physician-guided EOL MDM. Thus, the DM process was classified based on the process that was followed, not on the initial discussion about which process to follow.

### Guiding values for areas of the spectrum

**Fig. 3:**
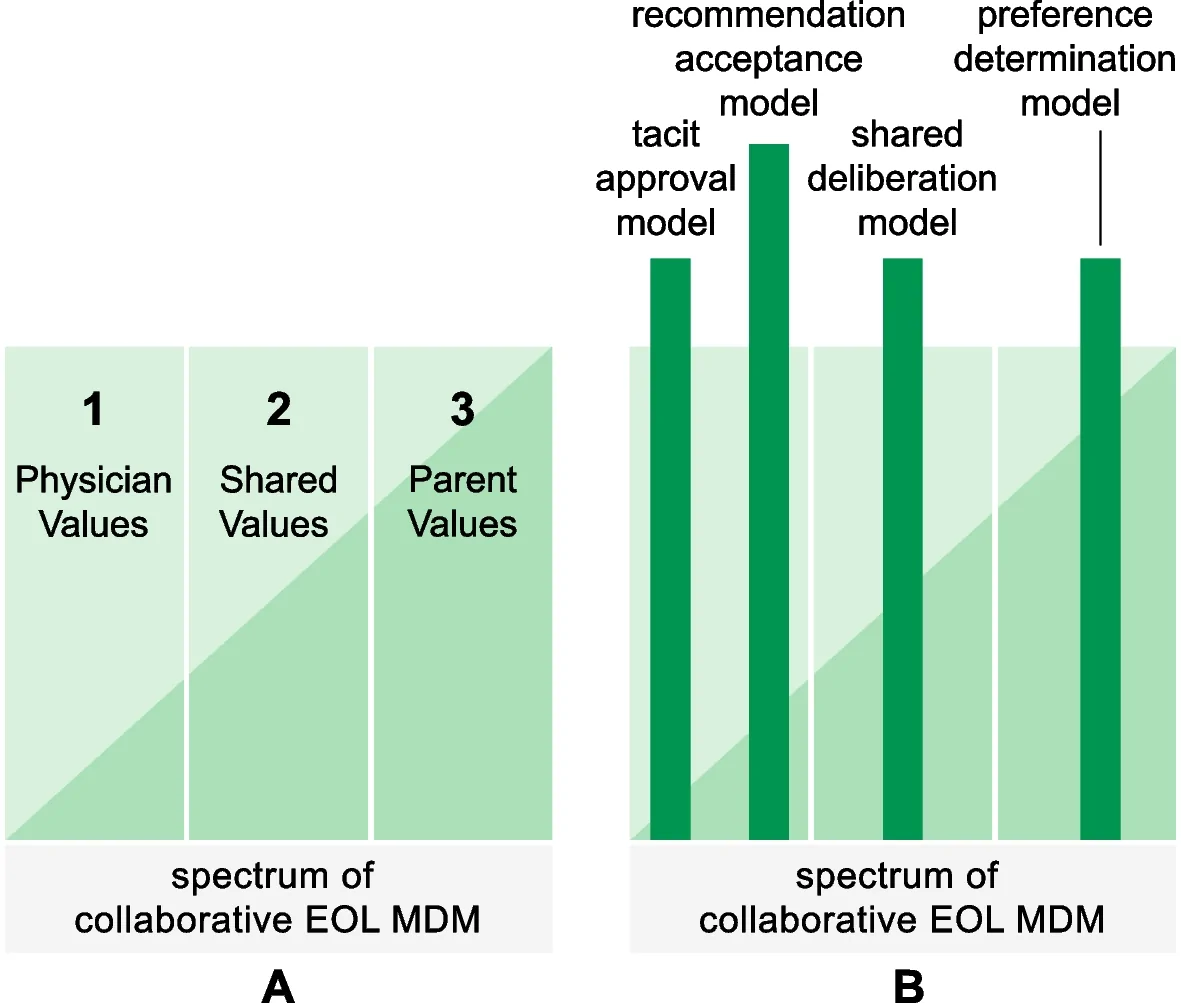
Guiding values and practice models for cEOL MDM. Legend: Fig. 3 consists of two panels (A and B). Panel A illustrates the distribution of the guiding values for cEOL MDM and Panel B presents different practice models on the spectrum of cEOL MDM. **A:** In area 1, the physician's (or medical team’s) professional values guide MDM. In area 3, parent values, which could incorporate health-related values, guide MDM. In the middle (area 2), both the parents' values and the physicians’ values guide MDM. **B:** The tacit approval model and the recommendation acceptance model are located within the left area, which represents parent-informed physician-guided MDM. The preference determination model is located within the right area, which represents physician-informed parent-guided MDM. The shared deliberation model is located within the middle area, which represents MDM with equal participation

The three areas of the cEOL MDM spectrum are further distinguished by the values that guide the process (see Fig. 3, panel A).

In *parent-informed physician-guided MDM*, it is the professional health-related values of the physician that guide the process (e.g., their evaluation of the child’s suffering, the determination of the child’s well-being). Here, we are not referring to the personal values of healthcare professionals ("What would they want if they were in the same situation as the parents?") , but rather to their values concerning the patient’s (often the child's) best interests (“What is best for this child under the circumstances?”). In *physician-informed parent-guided MDM*, it is the parents' or family's values that guide the DM process. These may furthermore include the parents' views of a child's purpose in life, what it means to care for a child with disability, or faith-based values such as accepting "fate" or "God's plan". In *MDM with equal participation* the guiding principles are those from both sides that were discussed and agreed upon by both parties.

### Practice models for cEOL MDM

The term "practice model" refers to a structured process for parental involvement. We identified four practice models which we located in the three areas of the spectrum of cEOL MDM (see Fig. 3, panel B). Each consists of specific sequential steps and includes a specific distribution of roles between the physician and parents [51, 52]. Even at the same NICU, different parents can experience different practice models and during their child's treatment, different practice models may be practiced at different times. Practice models can either be proposed by physicians, or parents.

The following practice models were identified: the tacit approval model, the recommendation acceptance model, the shared deliberation model, and the preference determination model. The articles by Caeymaex et al. [40] and Hendriks et al. [23] were most influential in the development of our understanding of these four models. Only the study by Caeymaex et al. described all of them at once. When studies referred to practice models as SDM, we translated them into the four models. We placed them on the spectrum according to how much parental involvement they entail. We explain them now in detail.

### Tacit approval model

We identified the tacit approval model in a variety of publications and it [23, 35–37, 40, 43, 45–47] was sometimes referred to as "medical decision-making" [40], "medical authority" [37] or "medical paternalism" [37]. We categorized it within the area of *parent-informed physician-guided MDM*as the model with the lowest parental participation.

In the tacit approval model, the decision authority and control lie exclusively with the physician. The physician determines which option is in the child’s best interest and then implements the treatment while constantly informing the child’s parents [37]. This is clearly different from MDM without parents, where the physician makes a unilateral decision without informing the parents.

In EOL MDM based on this model, physicians might inform the parents of the child’s current condition and determine intensive care as futile or they may share medical information with the parents, and explain how current and future impairments will affect the child's risk of mortality or of having a poor quality of life. The physician then might suggest forgoing LST as the only option because there are no realistic alternatives [37]. The informed parents might then approve of the proposed course of action by not objecting. In cases of disagreement, the implementation of the decision is postponed for a few days [37].

After experiencing the tacit approval model, parents usually do not remember actively participating in EOL MDM. In some cases, they don't even feel as though they have participated in a decision concerning the treatment of their (unborn) child and in other cases, they don’t even recall that a decision has taken place [23, 35, 37, 40, 43, 45–47]. This can stand in contrast to notes in medical files and reports of health care professionals stating that various options had been discussed [43].

This model was described in detail in the article by Orfali et al., which compared three units (A–C) in two countries: France and the USA. In French Unit A, one of the parents describes this model as follows: "Their discourse was quite clear. It was quite precise, there was no doubt over the situation. The physician didn’t really ask us what we wanted. No, nothing like that. Well, I don’t think anyway there was a choice to be made because you see, the brain damage was too big. There wasn’t any chance that he would get better. No. The way they told us, it was very accurate, only facts, you see…that you could understand without interpretation…". [37].

### Recommendation acceptance model

We identified the recommendation acceptance model in many publications [23, 35–41, 43, 47] and the authors referred to it by the "assent model" [41], "informed consent model" [46], "ethical decision making model" [23] or "not full parental involvement" [37]. We used the phrase "recommendation acceptance model" because the purpose of the conversation is not to reach a decision through an open-ended process, but rather to convince parents to accept the recommended treatment. We positioned it at the right end of *parent-informed physician-guided MDM* because here professional values of the physician still largely guide the DM process. Even if parents are actively asked to agree or disagree with a proposed action, their participation is limited. If they express disagreement with the proposed option, yet their veto could be decisive in determining the course of action.

In the study by Hendriks et al., the standard approach of the investigated unit in Switzerland was the recommendation acceptance model [23]. According to this model, the physician discusses the benefits and risks of intensive care with the unit’s medical or interprofessional team, resulting in a proposed treatment strategy (or recommendation). Then the physician explains the strategy to the parents and asks for their consent. The decision is primarily based on medical facts, the physicians’ or team’s professional experiences, and what they consider to be in the child’s best interests. The parents' values are determined indirectly and are not openly discussed with them. The physician provides parents with comprehensive medical information, enabling them to understand their infant’s health condition, the associated health risks and the proposed treatment strategy of the medical team (e.g. curative, palliative or watch-and-wait). Parents are then invited to ask questions and are subsequently asked to give their consent to the proposed course of action. A father said later: "What was really to decide? But actually…we relied on the staff and their advice. They have more experience, when they say it does not make any sense, then that was the decision. There was not really anything to decide." [23].

### Shared deliberation model

The shared deliberation model refers to a collaborative open-ended DM process conducted by both parties. Unlike the two other models it does not involve suggesting a predefined decision before the conversation between the physician and the parents. We only identified in two publications [40, 47]. This model was described in the article by Caeymeaux et al. as the "shared model" [40]. We position the shared deliberation model within the area of *MDM with equal participation*.

When structuring EOL MDM according to this model, physicians initiate conversations and empower parents to engage in the process. They start the conversation by providing information about the infant’s condition and the associated health risks and then they engage parents in a discussion about the relevant medical information, the available treatment options, the goals of health care, and the prognosis. Both parties contribute significantly to the DM, with the medical team expressing or affirming health-related values, and parents expressing parental or family values. Treatment preferences may then be expressed or commented on by both parties. Physicians can help parents weigh different options. A decision is reached when both agree on the proposed course of action. A parent involved in the French article by Caeymaex et al. described it as follows: "The doctor said to me, ‘your opinion is of course important, and your decision will be equally important, but you should know that the medical team also has an opinion and a decision." [40].

### Preference determination model

The preference determination model implies a division of tasks and responsibilities: While the physician is responsible for explaining the medical situation and possible treatment options, parents are responsible for evaluating the options and making the decisions. This model has been described in most publications [23, 35, 37–42, 45–50] and has been referred to as the ‘ neutrality model’ [41], ‘parental autonomy’ [42] or ‘informed parental decision making model’ [40]. We located it in the area of *physician-informed parent-guided EOL MDM*.

The model entails at least two conversations. In one conversation, the physician explains the (unborn) child’s condition, potential health risks, possible treatment options, their respective goals, and the presumed prognosis. In the other, parents are asked to consider the options in light of their interpretation of the infant’s best interests and their family values, but to do it in private, e.g., in a conversation with relatives, friends or other confidants [40]. Once parents have made their decision, they inform the physician in a second conversation.

This process is perceived as enabling parents to make self-reliant decisions, as expressed in the study by Baughcum et al. "They didn’t try to force their opinion one way or the other. They wanted it to be our decision and what we wanted to do. They provided us with the information, but let us go from there". [48].

### Evaluation of experience with cEOL MDM

Parents can evaluate their experience with cEOL MDM initially and retrospectively. Initial evaluation takes place during the process of DM, often shortly after conversations where a DM model is being proposed, while retrospective evaluation takes place later, after the DM process is completed.

### Initial evaluation

Initially, parents may evaluate their experience with EOL MDM either as consonant, if it meets their expectations, or dissonant, in case there is a discrepancy. When experiencing consonance, parents feel psychologically comfortable and experience positive feelings such as relief, appreciation, and well-being [35, 37, 39–41, 46, 47]. In the study by Kavanaugh et., for example, participants expressed immediate relief after the experience, as they were involved in the DM process as expected and were able to take responsibility for EOL-MDM: "So, talking to them made me realize, 'Okay, I am in charge' […]. So, I did feel better when they gave me options and choices because sometimes they were just like, 'Okay, you are going to do this and you are going to do that.'"[39].

Following a dissonant experience, parents may experience psychological discomfort and negative emotions such as helplessness, abandonment, fear, horror, pressure, bias and alienation [23, 36, 37, 39–41]. Once, parents felt abandoned because contrary to their expectation they were expected to make the decisions themselves: "They gave the choice to us, and it was difficult because they left us all alone, they left us really completely alone" [40]. Parents who experienced the tacit approval model in one study described their evaluation when their request to join the DM process had been turned down as follows: "It was an awful experience, that no one asked me what I wanted to happen. I remember asking the doctor whether we had any say, if they were going to ask us what we felt. 'No' they said, they never included the parents in these decisions; there was nothing to discuss. At that point of time I wanted them to do anything that would save my child. I didn’t manage to think about whether or not she would have a good life, that there was perhaps nothing to save her for. I would have saved her at any price, but we were not given the choice." [36].

### Retrospective evaluation

In retrospect, parents may evaluate their experience, including their role in the cEOL MDM, as either coherent or incoherent.

If parents retrospectively perceive their experience as coherent [23, 36, 37, 39–41, 48], they are able to identify a connection between themselves and their experience. Despite all the despair they might have experienced, the experience with cEOL MDM has meaning for them, and they may express gratitude for this realization. In one study, parents appreciated that they were able to fulfil their parental responsibilities: "We are her parents and we should make this decision. And we should decide what is best for our baby. Now in retrospect, I regard that as a great act of love." [23].

Alternatively, parents can retrospectively evaluate their experience of their role as incoherent [37, 40, 41, 48]. In one study, parents expressed difficulties with integrating their experience into their life story. One parent said: "We are the ones who said, then, on such a day, we stop. It is difficult for parents to tell themselves that they are not (well let’s say) 'killing' our child; it is that you stop, we stopped what was keeping her alive. You hate yourself." [40]. Some parents then think about the events contrafactually as that they should have opposed the proposed decision [40].

### Concordance and discordance between initial and retrospective evaluation

A retrospective evaluation can be similar or different from an initial evaluation. We use the term "concordance" when the retrospective evaluation aligns with the initial evaluation, and "discordance" when they differ. In most studies, the initial and retrospective evaluation were concordant evaluations [23, 37, 40, 41]. In discordant evaluations, for example parents initially evaluated their experience as dissonant but coherent in retrospect, initially feeling uncomfortable about their role in the process, but may later stating it made sense after all. In a one study, there were participants who initially felt uncomfortable about being excluded from the DM process, but later felt relieved that they did not have to take responsibility for the decision: "It was an awful experience, that no one asked me what I wanted to happen. […] Now, afterwards, […] I lean towards thinking that it was best that we were never asked."[36]. There is a theoretical possibility of the other type of discordance, yet, we did not find an example in the selected literature.

### Informed preference: parents’ preference for their role in cEOL MDM

Parents' preferences for cEOL MDM depend on their experiences. They have in principle two options to describe their preferred role: They can either describe how they would have liked their role to be in the past, or how they would prefer it to be in a similar situation in the future if they were again confronted with a decision about EOL MDM for a child. In a similar situation, they could express a willingness to repeat the DM process if their past role aligns with their future preferences, or they could request a change if their past role should not be offered again.

### Willingness to repeat

Several parents appreciated both their own role as well as the physician’s role in the DM process. In one study, a parent who experienced the tacit approval model was grateful: "I can’t say more than that I think it was a good thing that we were spared from making a decision. When I asked whether we had any say, I was turned down. I think perhaps it was good that someone made a decision, but I didn’t think so then and there. Now when a doctor says this or that, I’ll buy it. I won’t sort of discuss with a doctor the whys and wherefores." [36].

A parent in another study who experienced the recommendation acceptance model appreciated being asked for their agreement with the decision: "It is good that they ask you… that your opinion is valued" [37]. Parents who experienced the shared deliberation model appreciated feeling supported in taking an active role in the DM process [40]. A mother said: "The doctor said to me, ‘your opinion is of course important, and your decision will be equally important, but you should know that the medical team also has an opinion and a decision.’ That was good. I said to myself, Thank god, it isn’t me who has to decide. Because I had just been thinking what a real, total fright it would be to decide alone." [40]. Parents who experienced the preference determination model noted that, although it felt burdensome at the time, it was the right thing to do in the end: "He [the physician] said to us that the team would follow us in our decision, that he thought it was a good decision. That was great solace, because in fact it was our decision to make, and it was horrible to decide. At the time, it was great to be able to decide, that is, if a doctor had said to me, ‘We are deciding this’, it would have been unbearable for me. Here, it was difficult but the fact of having support, and hearing that … yes it did me a lot of good." [40]. Other parents who had experienced the preference determination model appreciated it because they felt prepared by the physician to fulfil their role. A mother said: "When it happens, you’re all mixed up, you just don’t know… Somebody has to help you, has to tell you: ‘These are the facts. You decide, and this is what you have to decide about’. For me, that was important." [41].

### Change request

Some parents may be dissatisfied with their and the physician’s roles in DM and may express a preference for a different role-allocation in the future [36, 37, 40, 46, 47]. The change may be a demand for either greater or lesser participation in DM [40, 42, 46].

Some parents who were in favor of less involvement in EOL MDM stated that they had more confidence in physicians than in their own DM abilities [36, 37, 40]. Others expressed that rather such a decision should be made by experts, e.g. because they felt overwhelmed when being offered the opportunity [36, 37, 40]. In one study, parents, who had experienced the preference determination model, felt left alone with a decision that was too challenging for them. A father said: "The doctors say to us: ‘It’s your choice. We are leaving the decision to you’. And finally, that is very, very hard. I don’t think it is a good thing" [53]. One mother felt she had no control over the decision because she did not have the means to keep her child alive: "I did not perceive that I had control of this, because I am not the doctor, I am not the one performing the resuscitation, I feel that I don't have control of this…" [47].

Parents who advocated for a higher level of involvement saw EOL MDM as part of their parental responsibility [36, 47]. In one study, a mother, who had experienced the tacit approval model said: "If the decision belongs to the parents, yes, in this case, yes […]. It would have been nice to have been told that ‘in this case there are these risks and the decision is yours if you want to keep going or not’…" [47].

### Parents’ preferences for a certain DM model

The various articles reported experiences with different forms of cEOL MDM (see Table S2 in Appendix 3). In only one publication, we identified all four models, although in some cases our interpretation differed from that of the authors [40]. This study involved data gathered in four hospitals across France up to three years after postnatal EOL MDM took place. Of the studies in which participants were recruited through specific organizations, the majority of these organizations appeared to have implemented more than one model [23, 35, 37–41, 43, 46, 47, 50]. In one case, the authors’ interpretation of interviews with the mother and father of a child showed that parents had different impressions of their experience, leading to the identification of two different practice models in the same case [35]. While the father thought the child's death was inevitable, the mother believed she had options and that choices had to be made.

We were unable to identify any models specific to Europe or North America. In an inter-cultural study, researchers were able to distinguish the micro cultures of a single organization from the cultural attributes associated with different countries [37]. Contrary to the prevalent notions of autonomy in the USA and paternalism in France, the authors found diverging patterns in the facilities under study. For instance, in both countries, parents described experiences with the recommendation acceptance model, but there were differences of its application between two NICUs within France (one offered it in cases of disagreement while the other didn’t).

The most common practice models in the sample were the preference determination model [23, 35, 37–42, 45–50], followed by the recommendation acceptance model [23, 36–41, 43, 46, 47] and the tacit approval model [23, 35–37, 40, 43, 45–47]. Despite its prevalence in the theoretical literature, experiences with the shared deliberation model has only been described clearly and in detail in the study by Caeymaex et al. [40]. In the study by Daboval et al. it is possible, yet not fully clear whether a shared deliberation took place [47]. A couple stated that "I’m glad that we do have a [a part in the] decision, because when it comes down to it, it is our baby, but also the fact [...] that we get the professional side of it as well." (Father—System #1) and "Even if we say yes, go for it, they won’t just do it and that’s all, they are going to take into consideration the baby’s health also." (Mother—System #1) [47].

Parents with experience in that regard preferred to be involved in EOL MDM, but the extent of their preferred involvement can vary greatly. In most cases, parents described their experience as coherent, regardless of its type and prefer the same approach to EOL MDM as the one they experienced. Parents who experienced dissonance with the role of DM that was offered to them [23, 35–37, 39–42, 46, 48, 50] sometimes either preferred more guidance from physicians [35–37, 40–42, 46, 48], and other times preferred less [23, 36, 39–41, 47, 50] (see Table S3 in Appendix 4).

## Discussion

This is the first QRS that presents a synthesis of the qualitative literature about the preferred role of parents in perinatal EOL MDM based on their experience with such a process. The study critically examines whether parents of (unborn) children prefer SDM over other approaches to EOL MDM. We found that their preference for a particular role in EOL MDM is based on previous experiences, processes of dissonance reduction and meaning-making. Different parents express different preferences regarding their involvement. The preferences of parents generally remain consistent over time. Only a few parents later prefer a different DM model, wanting either more or less involvement than experienced. Therefore, the claim that parents preferred SDM to other EOL MDM approaches has not been confirmed if SDM demarcates a specific form of cEOL MDM. It has only been confirmed if SDM is used as an umbrella term for at least three of the four described DM models through which parents and physicians collaborate: the recommendation acceptance model, the shared deliberation model and the preference determination model.

SDM is increasingly called for in perinatology, especially in situations where decisions have to be made in the so-called "grey zone". Lantos emphasized in regard to such decisions the necessity of a shift in a physician’s role from simply providing information about a child's current condition and probable prognoses to supporting parents through shared deliberation by helping them with the clarification of their values [54]. Following Lantos, the physician’s new role should be that of a choice architect framing and shaping the decisions that fall within parents' discretion. Such a call for SDM has also been made by medico-ethical guidelines for the care of premature infants at the limit of viability in various countries [6–8, 10, 11, 55].

Conversation analyses show that such an understanding of SDM is rarely being implemented in European NICUs [56–58]. Similarly to the results of this ME of interview studies, they show a variety of DM models. Sometimes, different models are combined in the hope of meeting various requirements at the same time. Physicians in our conversation analysis at a German NICU for example suggested finding a decision together with parents, while at the same time seeking parents’ agreement for the intended decision to forgo LST. [57]. From the parents' perspective, this can be perceived as SDM, even if their involvement in the decision is very limited. Our interview study showed that even if they follow a physician's recommendation, parents may still perceive the decision to be theirs [59].

Why is the norm "physicians and parents should conduct SDM" not realized in practice so far? Research on this issue so far has mainly focused on the behavior of physicians as barriers to the implementation of SDM, and state that either the willingness or the ability to engage in SDM was lacking. A recent qualitative study in a US NICU revealed that physician communication was a far greater barrier to SDM than facilitator [60]. When the communication concerning the options was unclear or included complex medical terms, it caused confusion. Simply knowing that choices exist seemed not to be enough for parents to feel supported in understanding the different options and feeling empowered to make their decision. Parents reported that physicians in this study failed to discuss family values and communicated poor prognoses without acknowledging the need for parents to maintain hope. A previous QRS identified the lack of medical information provided to parents to inform DM, an inadequate communication of information, poor relationships with caregivers, a lack of continuity in care, and perceived power imbalances between HCPs and parents as barriers to SDM [17]. Physicians in the USA on the other side felt their formal training did not include communication training for SDM, which limited their ability to involve parents in the DM process [43].

Physicians may be influenced by their perception of the legal and ethical status of a fetus versus a born child, offering different DM models according to whether the child is before or after birth. After birth they might be strongly influenced by their medico-ethical obligation to protect the child’s best interests. Some physicians might then understand SDM as a process where physicians might encourage parents to adopt their viewpoint [61]. This could entail then, depending on the situation, that physicians may opt for certain DM models, based on the medical situation and the likelihood of a fatal outcome or serious complications. When burdensome treatment outcomes are highly likely, they may feel a strong sense of decisional responsibility [17]. Physicians are very aware of their responsibility to do no harm, which is why, in situations where continuing LST would cause suffering, they may be inclined not to share DM authority with the parents [10, 20]. If however a positive outcome was highly likely, physicians might initiate LST in the child's best interest with less parental involvement [62]. They may assume that the parents had the same goals and would agree with their decision. However, in situations where doctors anticipate a potentially unfavorable outcome but are uncertain about the appropriate course of action, they may be more willing to engage in SDM, as they recognize that they must work together to understand the family’s values and incorporate them into the DM process [62]. Then, physicians might use conversations to explore the parents’ willingness to help their child cope with potentially burdensome health impairments. However, assessments of the prognosis vary depending on the physician. While they may agree to withhold treatment at the border of viability, physicians show significant variation in their approach to interpreting risks for other extremely preterm infants [63]. The parents' emotional crises could also influence their choices of a certain DM model. Physicians may not want to burden patients with EOL MDM if they are overtly distressed [17, 63].

Our study contributes to existing literature by demonstrating that the implementation of SDM depends on parental preferences. Parents do not prefer a single DM model, but different parents prefer different models of participation. From a parent's perspective, greater participation is not necessarily better, but rather the fit between parents’ needs and preferences with the process of DM seems crucial. Such an approach has been called a "tailored" or "personalized approach" to DM [21, 64]. Yet our study also highlights that the preferences prior to the situation might not be decisive in what parents consider to be a good DM process, as there were reports where initially the parents perceived the DM process as wrong but in retrospect they perceived it as right [36]. On a more general level, the nature of parental preferences for DM are relevant. Preferences for surrogate DM could be a personality trait (e.g., related to avoidance or acceptance of dependence on others) or a state that is influenced by contextual factors. Such factors occur on a macro-level (e.g., legal or healthcare systems, socio-cultural environments) [65], meso-level (e.g., healthcare environment implementation, physician leadership style) [66], or micro-level (e.g., personal factors such as distress) [67].

A prerequisite for a perception of a good or right DM approach is trust. In a QRS by Rosenthal and Nolan (2013) the researchers pointed out that trust in NICU health providers was essential for parents to be able to make decisions for their newborns. The ethical DM process begins when parents receive information and communicate with providers. The freedom with which information is provided and how easy and frequent communication occurs impacts parents’ trust in providers. Their level of trust also affects how they process the information they receive, how much further communication they require, and consequently, their level of engagement in the DM process. However, the perceived trust does not necessarily lead to a preference for a specific model. Rather, it is the parents' expectations, and their retrospective assessment of how well the experience aligns with these expectations, processes of dissonance reduction and of meaning making, that lead them to prefer a particular model.

### Implications for practice

Based on our findings, we suggest that physicians should be tailoring the right approach to DM based on a comprehensive analysis of the situation where expressed preferences are not the only factor in deciding about an initial DM approach. Such a proposal is consistent with a theory of family-centered care in pediatrics where the authors describe four distinct approaches to the physician-parent-relationship that differ with regard to leadership of the exchange (physician and/or parents) and degree of interaction (low or high): directing, teaching, collaborating and supporting [68]. They suggest a model of Adaptive Practice, where physicians adjust their style of family-centered care to the situation at hand, being mindful of the knowledge, experience, motivation, psychological state, culture and style of the parents. For example, in an unprecedented emergency situation directing the parents would be the most appropriate strategy where in situations where parents had substantial knowledge about their child’s illness it was justified to support them to do their own DM. According to this theory, conflicts arise when the physician and the parents are not synchronized about the approach. Although this theory was not specifically intended to guide DM in neonatal intensive care, it inspired our approach to *adaptive* cEOL DM, as it encompasses a range of DM models and recognizes the important role of parents' expectations in the DM approach.

A better understanding of our approach can be achieved by considering the communicative actions related to the different models. Table 1 shows how the deliberate choice of one of the four different models can be connected to possible communicative openings in the DM process. An explicit opening is essential for transparent communication, as discrepancies between parents' expectations and the actual process can cause dissonance. If a physician signals the model being used before the conversation begins, parents are given the opportunity to understand, accept, modify and reflect on the structure of their own participation in the DM process.

**Table 1:** Distribution of participants’ responses to the Generalized Anxiety Disorder-7 items among obese participants

DM model	Role allocation between parents and physician	Exemplary opening statement	Rationale for choice	Parents‘ possible recollection of the DM model
**Tacit approval model**	**The parents** receive information and implicitly approve by not objecting **The physician** determines the course of (further) treatment, taking into account the clinical condition of the (unborn) child	*My (or our) job is to make the best possible medical decisions for your child. I want to keep you fully informed about what is happening and what we are doing. I will explain everything, and you can ask me any questions you have at any time. *	- signals physician takes responsibility for the course of action - invites questions and fosters understanding	**consonance and/or coherence:** - may initially feel protected from burden of choice - may later feel relieved they were "spared" **dissonance and/or incoherence: ** - may not even remember that a decision was made - may have felt excluded from DM process - may have felt they had little control over their child’s care
**Recommendation acceptance model**	**The parents** receive a clear recommendation and are asked to give (or withhold) consent **The physician** makes recommendations for further treatment, taking into account the clinical condition of the unborn child, as well as the values and preferences of the parents	*Having reviewed everything (with our team), ) (we) believe that the best course of action for your baby is as follows. I would like to talk you through our reasoning and find out whether you and your family can agree with this approach.*	- makes recommendation explicit and transparent - creates space for discussion without the need to participate in the whole DM process - asks parents to give their consent or to express their objection	**consonance and/or coherence:** - may feel heard and respected - may appreciate the clarity of recommendation - may recall that the parents made the decision themselves **dissonance and/or incoherence:** - may feel the "real" decision was already made before the conversation -may have “signed off” a decision that they did not take
**Shared deliberation model**	**The parents** and **the physician** contribute equally to the open discussion of treatment options and family values before a final decision is reached	*There is no one-size-fits-all answer here, and your/ your family’s values play an enormously important role in the decision-making process. I’d like us to think this through together – I’ll explain what the medical evidence says, and I’d like to hear from you what matters most to you.*	- indicates that no decision has yet been made - presents the decision-making process as a joint endeavour	**consonance and/or coherence: ** - may feel supported and not alone -may feel reassured to be on the right path because the physician also has a view **dissonance and/or incoherence:** - may feel at a disadvantage during the conversation -may feel guilty for being part of the decision
**Preference determination model**	**The parents** receive medical information and then make the decision themselves, typically during private reflection **The physician** gives information about the clinical situation of the (unborn) child, the treatment options and then asks for the parent’s decision and implements it	*We will provide you with all the information you need, and ultimately the decision is yours. We will carry out your decision and support you every step of the way.*	- indicates that the decision has to be made by the parents outside of the physician-parent conversation - strengthens parental authority and provides the best evidence for their deliberation	**consonance and/or coherence: ** -may feel empowered and responsible - may see their active role as a meaningful act of parental love **dissonance and/or incoherence: ** - may feel overburdened and abandoned -may feel the full weight of life-and-death decisions on their shoulders

Further normative-ethical analysis is needed to inform deliberations about which DM model is ethically legitimate in specific situations. The central research question for such studies is therefore: Which model for cEOL MDM should be used in a particular situation? To answer this question, we first have to clarify: Which ethical norms are relevant with regard to decisions as such? Such a study could result in a normative framework to guide cEOL MDM.

Furthermore, our study results have implications for the construction of guidelines for EOL MDM in the NICU [6, 7, 10, 69]. The German medico-ethical guideline, among other international guidelines [6, 7, 10, 69], has recommended SDM in EOL MDM in perinatology, particularly in situations where the prognosis is uncertain but potentially poor [3]. It was argued that parents preferred SDM over other forms of cEOL MDM. However, based on our QRS, we cannot confirm that all parents prefer SDM to other forms of cEOL MDM if it relates to the model of shared deliberation. According to the analyzed studies only a small minority actually experienced SDM understood as a shared deliberation of treatment options and expected outcomes.

It has been argued that SDM is prescribed in clinical practice guidelines [4, 65, 70]. Such guidance potentially violates bioethical norms and physician’s legal obligations [71]. In light of the expectable variability in the context under which such decisions are to be made the suitable model for a certain DM situation might better be determined during the actual encounter and not in a guideline. When guidelines recommend SDM they should specify how they define the term. Otherwise, their recommendations can neither be implemented nor evaluated. Physicians who work in perinatology should be provided with training to this effect. They should be proficient in all four models and be able to deliberately and appropriately implement one of them in a specific case.

### Limitations

This QRS has several limitations. We restricted our literature search to three databases and the selection of included articles was limited to publications in English, and to studies conducted in North America and Europe. The included study contexts vary greatly with regard to laws, health systems, cultural orientations and practice standards. The abstracts were reviewed by one person before the full-text articles were obtained and relevant publications may have been overlooked.

We investigated a limited set of research questions, which we selected based on our interest in medico-ethical guidelines for EOL MDM in perinatal care. There are further relevant questions that other researchers could investigate with the identified sample of studies, for example "What contributes to parents’ appreciation or disapproval of the DM process?" Some of the cited quotes suggest that feeling heard and being acknowledged as capable decision-makers is key to a positive evaluation of the DM process. Gaining more insight into such parental preferences could give physicians a broader perspective on how to approach EOL MDM.

Our inclusion criteria were narrow and may have led to the systematic exclusion of relevant publications. As we have focused on the parents’ perspective, we mostly report on their interpretations of the DM process. We only included qualitative interview studies with parents who had *experienced* EOL MDM in perinatology. Through our literature search, we did not identify any prospective longitudinal studies that investigated parents' expectations prior to EOL MDM. We did not include interviews on hypothetical scenarios in which preferences were investigated without EOL MDM taking place [16]. Furthermore, we did not systematically include natural data based on physician-parent conversations [23, 56, 57], nor qualitative studies solely concerned with the perspectives of physicians or other team members [72]. In some cases, the publications we analyzed incorporated data obtained from physicians and other healthcare professionals working in NICUs through interviews or medical charts. Oftentimes, the research teams incorporated healthcare professionals who had considerable experience working in the NICU as physicians or nurses. As a result, it is possible that, in describing the sphere of cEOL MDM, the areas of MDM and the four models, we also drew on knowledge that did not originate solely from parents but also from physicians and nurses with work experience in perinatal care. Yet, while some descriptions may overlap in studies involving parents, physicians and other team members, the theory of parental preference determination can only be developed from studies focusing on parental preferences. Further analysis of studies with professionals could, as a form of triangulation, help to validate our findings across different datasets [73].

Due to limitations in data selection, our theory may be incomplete and could be further developed by considering additional factors. For example, none of the studies in our sample described EOL DM without collaboration between physicians and parents. This is due to the studies’ recruitment strategies, in which participants were approached through institutions where DM processes took place.

Since the majority of the articles were framed through the perspective of health professionals with experience in neonatal care, our theory could be further enlightened by the insights of researchers from other fields. For instance, our theory of cEOL MDM could be expanded by reflecting on how the professional values of physicians and other healthcare professionals are formed. Professional values are not "objective" and may be influenced by personal beliefs relating to quality of life, a child's suffering or EOL MDM. For instance, this has been acknowledged in a theory of professional identity formation of physicians [74].

All participants were interviewed following the DM discussions. Half of the studies included parents who were invited to participate a considerable amount of time after the DM process had taken place, ranging from two months to eight years [23, 36, 37, 40, 42, 43, 48, 50]. Parents may have selectively recalled certain aspects of the experience. There could be many reasons for this, one of which is the process of rationalizing some aspects in order to make sense of the experience. Combining conversation analysis with interview analysis could be a promising future study approach for improving our understanding of such processes. None of the included articles focused exclusively on the question of preference formation, therefore we neglected other important issues reported in the articles. Lastly, preference research in general could be biased as dissatisfied parents could be less willing to share their experiences with researchers, especially if -and that is most often the case- the study was conducted by the institution they are dissatisfied with.

## Conclusion

As adopted DM models, and consequently the level of parental involvement in perinatal EOL-MDM, as well as parents' preferences, vary considerably across different contexts, it is questionable whether guidelines should prescribe only one DM model. Rather than aiming to give parents maximum DM authority, physicians should adapt their choice of model to the specific situation. While parents' expectations are one factor in deciding on the appropriate model, other factors, such as the infant's prognosis, also need to be considered. Physicians should receive training in effective communication for cEOL MDM to adapt their behavior to the situation at hand.

## Supplementary Information

Supplementary Material 1

Supplementary Material 2

Supplementary Material 3

Supplementary Material 4

## Data Availability

All data supporting the findings of this study are available within the paper and its Supplementary Information.

## Abbreviations

EOL MDM: End-of-life medical decision-making
LST: Life-sustaining treatment
SDM: Shared decision-making
DM: Decision-making
MDM: Medical decision-making
cEOL MDM: Collaborative EOL MDM
ME: Meta-ethnography
NICU: Neonatal intensive care unit
