# Supplementary material for: How parents derive their informed role preferences for end-of-life medical decision-making for (unborn) children in perinatology: a meta-ethnography of qualitative interview studies
Source: BMC Med Ethics. 2026 Sep 18;27:199. doi: 10.1186/s12910-026-01605-1 (PMC13589376; doi:10.1186/s12910-026-01605-1)
Supplement: Supplementary file 1 [file 12910_2026_1605_MOESM1_ESM.docx]

Appendix S1: Search term for the systematic literature search with MEDLINE via PubMed

Date last searched: 30.11.2023

(((((NICU*[TITLE/ABSTRACT] OR «ICU, NEONATAL»[TITLE/ABSTRACT] OR «NEONATAL ICU*»[TITLE/ABSTRACT] OR «NEONATAL INTENSIVE CARE UNIT*»[TITLE/ABSTRACT] OR «NEWBORN ICU*»[TITLE/ABSTRACT] OR «NEWBORN INTENSIVE CARE UNIT*»[TITLE/ABSTRACT] OR NEONATOL*[TITLE/ABSTRACT] OR PERINATOL*[TITLE/ABSTRACT] OR «NEONATAL PREMATURITY»[TITLE/ABSTRACT] OR «PREMATURE INFANT*»[TITLE/ABSTRACT] OR «PRETERM INFANT*»[TITLE/ABSTRACT] OR NEONATE*[TITLE/ABSTRACT] OR NEWBORN*[TITLE/ABSTRACT] OR PREMATUR*[TITLE/ABSTRACT] OR PRETERM*[TITLE/ABSTRACT] OR BABY*[TITLE/ABSTRACT] OR PREEMIE[TITLE/ABSTRACT] OR «INTENSIVE CARE UNITS, NEONATAL»[TITLE/ABSTRACT] OR «INFANT, NEWBORN»[TITLE/ABSTRACT] OR «INFANT, PREMATURE»[TITLE/ABSTRACT])) AND (FAMIL*[TITLE/ABSTRACT] OR PARENT*[TITLE/ABSTRACT] OR FATHER*[TITLE/ABSTRACT] OR MOTHER*[TITLE/ABSTRACT] OR «FAMILY MEMBER*»[TITLE/ABSTRACT] OR RELATIVE*[TITLE/ABSTRACT] OR «LEGAL GUARDIAN»[TITLE/ABSTRACT] OR PROXY[TITLE/ABSTRACT] OR «NUCLEAR FAMILY»[TITLE/ABSTRACT])) AND («PALLIATIVE CARE»[TITLE/ABSTRACT] OR «PALLIATIVE SUPPORTIVE CARE»[TITLE/ABSTRACT] OR «PALLIATIVE THERAPY»[TITLE/ABSTRACT] OR «PALLIATIVE TREATMENT»[TITLE/ABSTRACT] OR «THERAPY, PALLIATIVE»[TITLE/ABSTRACT] OR «TERMINAL CARE»[TITLE/ABSTRACT] OR «END OF LIFE CARE»[TITLE/ABSTRACT] OR «END-OF-LIFE»[TITLE/ABSTRACT] OR «EUTHANASIA, PASSIVE»[TITLE/ABSTRACT] OR «PASSIVE EUTHANASIA»[TITLE/ABSTRACT] OR «ALLOWING TO DIE»[TITLE/ABSTRACT] OR «PALLIATIVE MEDICINE»[TITLE/ABSTRACT] OR «MEDICINE, PALLIATIVE»[TITLE/ABSTRACT] OR «PALLIATIVE CARE MEDICINE»[TITLE/ABSTRACT] OR «WITHHOLDING TREATMENT»[TITLE/ABSTRACT] OR «WITHDRAWING TREATMENT»[TITLE/ABSTRACT] OR «WITHDRAWING CARE»[TITLE/ABSTRACT] OR WITHDRAW*[TITLE/ABSTRACT] OR «CESSATION OF TREATMENT»[TITLE/ABSTRACT] OR NONTREATMENT[TITLE/ABSTRACT] OR «LIMITATION OF LIFE-SUSTAINING TREATMENT»[TITLE/ABSTRACT] OR «LIMITATION OF THERAPY»[TITLE/ABSTRACT] OR «LIMITATION OF TREATMENT»[TITLE/ABSTRACT] OR «PATIENT COMFORT»[TITLE/ABSTRACT] OR «COMFORT CARE»[TITLE/ABSTRACT] OR PALLIATIV*[TITLE/ABSTRACT] OR WITHHOLD*[TITLE/ABSTRACT] OR «END OF LIFE»[TITLE/ABSTRACT])) AND («DECISION MAKING»[TITLE/ABSTRACT] OR «DECISION-MAKING»[TITLE/ABSTRACT] OR DECISION*[TITLE/ABSTRACT] OR DECIDE[TITLE/ABSTRACT] OR «SHARED-DECISION»[TITLE/ABSTRACT] OR «DECISION MAKING, SHARED»[TITLE/ABSTRACT] OR COMMUNICATION[TITLE/ABSTRACT] OR «HEALTH COMMUNICATION»[TITLE/ABSTRACT] OR CONVERSATION[TITLE/ABSTRACT] OR CONFERENCE*[TITLE/ABSTRACT] OR COUNSEL*[TITLE/ABSTRACT] OR INFORMATION[TITLE/ABSTRACT] OR MEETING[TITLE/ABSTRACT] OR INTERACTION[TITLE/ABSTRACT] OR DILEMMA[TITLE/ABSTRACT] OR DISCUSS*[TITLE/ABSTRACT]))
