# Supplementary material for: How parents derive their informed role preferences for end-of-life medical decision-making for (unborn) children in perinatology: a meta-ethnography of qualitative interview studies
Source: BMC Med Ethics. 2026 Sep 18;27:199. doi: 10.1186/s12910-026-01605-1 (PMC13589376; doi:10.1186/s12910-026-01605-1)
Supplement: Supplementary file 2 [file 12910_2026_1605_MOESM2_ESM.docx]

**Table S1 Characteristics of the original articles included in the review**

| **No** | **Authors (year)** | **Continent** | **Country/State** | **Design** | **NICU setting** | **Participants** | **Type of decision(s) for (unborn) child** | **Health issues** | **Data** | **Time points of interviews with parents** | **Data analysis** |
| --- | --- | --- | --- | --- | --- | --- | --- | --- | --- | --- | --- |
| #1 | Wocial (2000) | North America | USA/Minnesota | retrospective | 1 hospital/ NICU in large metropolitan area | 12 cases, 20 parents, 13 infants, all infants died | postnatal MDM:  forgoing LST | premature delivery, congenital malformation, acquired disease or damage | open-ended face-to-face or telephone interviews with parents; demographic data; medical charts | one time point between 4 to 12 months after the death of the child | qualitative content analysis |
| #2 | Kavanaugh et al. (2005) | North America | USA/Illinois | prospective | 2 hospitals that provided high-risk perinatal care | 6 cases, 8 parents (6 mothers, 2 fathers), 6 infants, 1 infant died;6 physicians, 2 nurses | pre- and postnatal MDM:  termination of pregnancy, pregnancy management, mode of delivery, resuscitation, forgoing LST | risk of premature delivery | face-to-face interviews with parents (separately or together); separate telephone interviews with physicians and nurses; periodic assessments; demographic data; medical charts | several time points between identification of risk for premature birth and up to 28 days after delivery, interview before birth, after birth and in one case 1-3 weeks after the infant’s death | qualitative collective case study; usage of the Ottawa Decision Support framework for coding and analysis |
| #3 | Payot et al. (2007) | North America | Canada/Quebec | prospective | 1 hospital, reference centre for at-risk pregnancies and neonatal care that is located in a perinatal tertiary care centre in Montréal | 8 cases, 16 parents (8 mothers, 8 fathers), 8 infants, 6 infants died;  4 physicians | prenatal MDM: resuscitation | risk of premature delivery | separate face-to-face semi-directed in-depth interviews with parents and physicians | two time points, one immediately (up to one day) after the antenatal consultation following the identification of risk for premature birth and 4-6 months after the consultation | hermeneutic and dialectical approach; qualitative data analysis according to the constant comparative method (constructivist Grounded Theory Methodology) |
| #4 | Boss et al. (2008) | North America | USA/ Maryland | retrospective | 3 hospitals, 2 urban regional referral centres, 1 suburban community hospital | 26 cases, 26  parents (mothers), 26 infants, all infants died | prenatal MDM: resuscitation | premature delivery, congenital malformation | telephone interviews and one and e-mail interview with a semi-structured instrument with mothers; demographic data; medical charts; | one time point between 10 months and 5 years after the infant’s death | coding of content, identification of key themes based on frequency |
| #5 | Kavanaugh et al. (2009) | North America | USA/Illinois | prospective | 1 hospital | 1 case, 2 parents (1 mother, 1 father) of 1 infant, the infant died, 1 physician, 2 nurses | prenatal MDM: LST maintaining or interrupting the pregnancy, removal of cerclage, bed rest with hospitalisation or at home | risk of premature delivery | separate face-to-face interviews with interview guides with mother/father; and telephone-interviews with a physician and nurses, demographic data; medical charts | one time point immediately after the antenatal consultation (the same day) following the identification of risk for premature birth | qualitative collective case study; usage of the Ottawa Decision Support framework for coding and analysis |
| #6 | Moro et al. (2011) | North America | USA/Illinois | prospective | 3 hospitals | 5 cases, 5 parents (mothers), 7 infants, all infants died; 4 physicians, 4 nurses | pre- and postnatal MDM:  forgoing LST | risk of premature delivery | face-to-face interviews with mothers; separate face-to-face or telephone interviews with physicians and nurses; periodic assessments; demographic data; medical charts | several time points between identification of risk for premature birth and following the death of the infants; interviews before birth, after birth and several weeks to months after the infant’s death | collective case study; analysis of interviews according to the Ottawa Decision Support framework, within and across case analysis |
| #7 | Kavanaugh et al. (2015) | North America | USA/a midwestern state | prospective | 3 hospitals | 40 cases, 54 parents (40 mothers, 14 fathers) | prenatal MDM: LST | risk of premature delivery | open-ended face-to-face interviews with parents; field notes | several time points; data gathered during the prenatal period | secondary analysis; directed content analysis using Swanson's middle-range theory of caring |
| #8 | Daboval et al. (2016) | North America | Canada/Ottawa | prospective | 1 tertiary care hospital/department of Obstetrics and Gynaecology in Ottawa | 5 cases, 10 parents (5 mothers, 5 fathers);6 infants,  5 physicians, 1 fellow in neonatal perinatal medicine | prenatal MDM | risk of premature delivery | face-to-face semi-directive in-depth interviews with both parents; separate interviews with physicians; satisfaction score;  demographic characteristics | one time point within four hours after the antenatal consultation | multiple case ethnomethodological qualitative research study; content and iterative analysis from a constructivist orientation |
| #9 | Baughcum et al. (2017) | North America | USA/ Ohio | retrospective | 1 hospital/ large midwestern level IV NICU | 31 cases, 45 parents (29 mothers, 16 fathers), 31 infants, all infants died | postnatal MDM:  resuscitation, forgoing LST | risk of premature delivery, congenital malformation, acquired disease or damage | open-ended telephone interviews; demographic characteristics; medical charts | one time point between 3 months and 5 years after the infant’s death | content analysis |
| #10 | Tucker Edmonds et al. (2019) | North America | USA/ midwestern | prospective | 3 tertiary care hospitals | 40 cases, 54 parents (40 mothers, 14 fathers) | prenatal MDM:  termination of pregnancy, pregnancy management, mode of delivery, resuscitation | risk of premature delivery | face-to-face interviews with interview guides with mother and father (separately or together) | one time point immediately after the antenatal consultation (the same day or the day after) | conventional content analysis |
| #11 | Denney-Kölsch & Côté- Arsenault (2022) | North America | USA/ Mid-Atlantinc and Midwest states | prospective | multiple centres in several Mid-Atlantic and Midwest U.S. states | 16 cases, 30  parents (16,  mothers, 13 fathers and 1 female partner), 16 infants, 14 died | prenatal MDM:  termination vs continuing pregnancy, prenatal care options, approach to care for baby at birth | congenital malformation, acquired disease or damage | in-depth face-to-face or telephone or video conference interviews with an interview guide with parents (separately or together) | several time points, up to four interviews, two prenatal interviews twice during pregnancy, twice after birth and death | inductive data analysis with two cycles of coding and theme identification, within and across case analysis |
| #12 | Orfali & Gordon (2004) | North America and Europe | USA/not specified  and  France | retrospective | three academic hospitals/level III NICUs, one urban hospital in USA, one urban and one suburban hospital in France | 85 cases,  75 parents (mothers); 60 physicians | postnatal MDM: resuscitation, forgoing LST | premature delivery, congenital malformation, acquired disease or damage | in-depth semi-structured face-to-face interviews with mothers, separate  in-depth semi-structured interviews with physicians, demographic characteristics, medical charts,  observational field work | in the US setting: one time point between 2 and 3 months after death; in the French setting: after family-staff meetings, after discharge or several weeks after the infant's death | comparative ethnographic research |
| #13 | Brinchmann et al. (2002) | Europe | Norway | retrospective | multiple centres throughout Norway | 26 cases, 35 parents (19 mothers, 16 fathers) of which 4 were health professionals, 26 infants, 10 infants died; 10 nurses, 1 physician | postnatal MDM: forgoing LST | premature delivery, congenital malformation, acquired disease or damage | in-depth unstructured face-to-face interviews with mother and father (separately or together), separate conversations and interviews with one physician and nurses | one time point between 1 and 8 years after the decision-making process | comparative method (grounded theory) |
| #14 | Caeymaex et al. (2011) | Europe | France | retrospective | 4 hospitals/ NICUs in different areas | 133 cases, 164 parents (103 mothers, 61 fathers), 139 infants, all infants died | postnatal MDM: forgoing LST | premature delivery, congenital malformation, acquired disease or damage | in-depth semi-structured face-to-face-interviews with a thematic guide or less structured telephone interviews with parents (separately or together), demographic characteristics, medical charts | one time point 2.8 years (+/- 0.7 years) after the infant’s death | discourse analysis; quantitative analysis |
| #15 | Hendriks & Abraham (2017) | Europe | Switzerland | retrospective | 1 hospital/ level III NICU | 13 cases, 20 parents (12 mothers, 8 fathers), 13 infants, all infants died; 1 physician, 1 physiotherapist, 1 psychologist, 1 pastor, 1 music therapist | pre- and postnatal MDM: pregnancy management, resuscitation, forgoing LST | premature delivery | narrative interviews and follow-up questions with an interview guide (face-to-face) with parents (separately or together), field notes from observations in the NICU, separate informal interviews with staff members, chat forums used by parents | one time point between 1 to 2 years after infant’s death | hermeneutically oriented research design, qualitative content analysis |
| #16 | Geurtzen et al. (2019) | Europe | Netherlands | retrospective | 6 tertiary care hospitals throughout the Netherlands | 13 cases, 20 parents (13 mothers, 7 fathers), 19 infants, 12 infants died | prenatal MDM: forgoing LST | premature delivery | semi-structured open-ended face-to-face interviews with an interview guide with parents (separately or together), demographic characteristics, investigator's field notes | one time point between 2 and 5 years after prenatal counselling | sorting interview data after themes and domains, choosing summarizing terms |

LST life sustaining therapy

MDM medical decision-making

NICU neonatal intensive care unit
