# Supplementary material for: How parents derive their informed role preferences for end-of-life medical decision-making for (unborn) children in perinatology: a meta-ethnography of qualitative interview studies
Source: BMC Med Ethics. 2026 Sep 18;27:199. doi: 10.1186/s12910-026-01605-1 (PMC13589376; doi:10.1186/s12910-026-01605-1)
Supplement: Supplementary file 3 [file 12910_2026_1605_MOESM3_ESM.docx]

**Table S2 Experienced forms of cEOL MDM**

| **No** | **Articles**  **(Authors/Year)** | **Area** | **Practice model** | **Examples for descriptions in the articles** |
| --- | --- | --- | --- | --- |
| #1 | Wocial (2000) | Physician-informed parent-guided MDM | Preference determination model | “Control emerged as one of the significant themes relevant to parents' level of involvement in the decision making process. Parents felt involved when they had a feeling that they were in control. They needed to feel they had choices and the power to exercise them. Without these, parents could not be parents.  “We said "Yeah, that is what we want." (withholding treatment)[horizontal ellipsis]. They were really nice about it. You know, they told us, you know, that at anytime we could change our mind and let them know and they would go ahead and do it (the treatment) or whatever, but they gave us that option[horizontal ellipsis].”” (p.83) |
| #2 | Kavanaugh et al. (2005) | Parent-informed physician-guided MDM | Tacit approval model | “In contrast, only one mother, who has a child who was born at 25 weeks’ gestation, felt that it was the physicians’ decision alone because of their expertise. She said, “They (physicians) want, they want to leave making my decision on what they do. But, I’m not a doctor, so it won’t be my decision. I’m trusting these guys with, with everything they do.”“ (p. 354) |
|  |  |  | Recommendation acceptance model | “This mother’s infant died and at the postdeath interview, she indicated that the end-of-life decisions were also not hers to make. She felt that the physician should just initiate any treatment that is medically indicated. She said, “I didn’t make any decisions other than giving them confirmation over the phone for them to change the two lines and initially when she first got the blood transfusions.” (p. 354) |
|  |  | Physician-informed parent-guided MDM | Preference determination model | “One physician explained his role: „I think your job is to really discuss the medical aspects of their situation and give them the best information you can. I think you have to be honest with yourself and your own degree of knowledge and get the best information to them that is available. Once you do that, you then try to support the decision they make.“” (p. 354) |
| #3 | Payot et al. (2007) | Parent-informed physician-guided MDM | Recommendation acceptance model | “In this model, the neonatologist’s preferences are clearly expressed and a decision is sought during the consultation.” (p.1490)  “Neonatologist 4  “I told them that we would not resuscitate at 23 weeks. So I think there wasn’t any specific contract to make with them, other than confirming what had already been said by the obstetrician, that we would not do any resuscitation.”” (p. 1490) |
|  |  | Physician-informed parent-guided MDM | Preference determination model | “They provide information, hoping that it will be well understood, to enable parents to make well-informed decisions.” (p. 1490)  “Neonatologist 2  “The importance of giving the most objective information, for me, is a reflection of the value that family has for me, its integrity, and the fact that this child is the child of these parents, not mine, not belonging to medicine. I believe it is up to them to know what is expected and what to do.”” (p. 1490) |
| #4 | Boss et al. (2008) | Parent-informed physician-guided MDM | Tacit approval model | “Despite desiring to participate in decision-making and having spoken with 1 physician about the high-risk pregnancy, few parents recalled discussing options for delivery room resuscitation. Even fewer recalled discussing compassionate care. “ (p.585)  “I think it seemed to me it was more so I was given this is what could happen in the NICU and what would probably happen. It was not so much a decision but more so information.” (p. 585) |
|  |  |  | Recommendation acceptance model | “Parents explained that what they needed most from health care providers was compassion and hope that the infant could survive. Women mistrusted physicians who communicated only negative information and seemed to have “given up.” (p.585)  “One physician kept wanting to induce me earlier and earlier. She kept saying this pregnancy is not a good one and stuff and shortly after the baby was born she came up to me and wanted to do an autopsy and that was not going to happen. I mean, had she let me grieve for longer than 20 minutes I might have let something happen as far as the autopsy, but I didn’t want that woman coming anywhere near my child at that point.” (p. 586) |
| #5 | Kavanaugh et al. (2009) | Parent-informed physician-guided MDM | Tacit approval model | “John did not remember that the physicians offered treatment options when Susan was admitted to the hospital, but he recalled that the physicians talked about the rupture of membranes and that they were sorry this had happened. Hearing this, John thought, “So it was kind of like the pregnancy was over then. (parent)” (p. 5)  “According to Susan, the physician told her that her infant would not be resuscitated until 24 to 25 weeks gestation when the infant would be viable.” (p. 5) |
|  |  | Physician-informed parent-guided MDM | Preference determination model | “Susan was hopeful she would make it to that point and was certain about her decision to keep the cerclage in and remain in the hospital on bed rest. According to Susan: “I am 22-1/2 weeks, which is 22 weeks and 4 days. So, they were asking me….I can either terminate the pregnancy or try to hold out until I am at least 24 weeks that way the baby can have like a 20% to 30% chance to make it….If the baby does make it, the baby still might have major problems. I told them I understand that and that I may be at risk of having some kind of infection….I’m 10 days from being at 24 weeks, so I am going to try my best to hold out. So, hopefully everything will work out for me.”“ (p. 5) |
| #6 | Moro et al. (2011) | Parent-informed physician-guided MDM | Recommendation acceptance model | “One of these physicians told the mother and grandmother that they had done all they could and the infant was suffering. After the discussion, the mother and grandmother gave permission for the physician to remove the ventilator from the infant.” (p. 4) |
|  |  | Physician-informed parent-guided MDM | Preference determination model | “In one case, the physician recalled that the mother wanted to wait for the father before making the decision to withdraw life support from the infant. In another case, the physician said that the mother received a lot of support from her mother, sister and brother-in-law and that her family was involved in making life support decisions.” (p. 5) |
| #7 | Kavanaugh et al. (2015) | Parent-informed physician-guided MDM | Recommendation acceptance model | “When providers respected the parents’ choices and did not impose guilt or pressure, even when parent decisions were not in agreement with provider advice or beliefs, parents felt the providers believed in them: “So, talking to them made me realize, “Okay, I am in charge,” and they give me that much respect to me to say, you know, okay this is what should be done and that should be done, because actually they do listen. So, I did feel better when they gave me options and choices because sometimes they were just like, “Okay, you are going to do this and you are going to do that.”.”(p. 148) |
|  |  | Physician-informed parent-guided MDM | Preference determination model | “Knowing begins by asking parents about their preferences, listening to what is said, tuning in to what is not said, and avoiding assumptions about what might matter to them:“They came and asked us like, they had asked us what do we want to do when the baby, if the baby comes early. Do they want, do we want the full attention as possible? And we was like “Yeah,” and they agreed with us; you know what I am saying, it was all about what we wanted and they was willing to help with anything.” (p. 149) |
| #8 | Daboval et al. (2016) | Parent-informed physician-guided MDM | Tacit approval model | “The parents who were not satisfied with their participation felt that the physician had all the control over treatment decisions. “I did not perceive that I had control of this, because I am not the physician, I am not the one performing the resuscitation, I feel that I don't have control of this. . .” (Mother - System #5)” (p. 6) |
|  |  |  | Recommendation acceptance model | “It is a parent decision notwithstanding physician’s opinion: “I understand that, but it is still my decision because it is my child, right?. . .,. . . I appreciate a doctor to say they do not think it will work, but you can still try. I am glad they have an opinion, but I have one too.” (Mother - System #2)” (p. 6) |
|  |  | MDM with equal participation | Shared deliberation model | “It is parent decision but physician may have an opportunity to make a different choice: “I’m glad that we do have a [a part in the] decision, because when it comes down to it, it is our baby, but also the fact [. . .] that we get the professional side of it as well. (father) [. . .] Even if we say yes, go for it, they won’t just do it and that’s all, they are going to take into consideration the baby’s health also.” (Mother - System #1)” (p. 6) |
|  |  | Physician-informed parent-guided MDM | Preference determination model | “It is a parent decision based on selected information: “If the baby was coming, I would give a chance to my child, I said it with my words. [. . .] The doctors know what is good for the baby. So we share the information and I triage them. I am taking what is good for my baby and me. “ (Mother - System #4)“ (p. 6) |
| #9 | Baughcum et al. (2017) | Physician-informed parent-guided MDM | Preference determination model | „parents as partners in care“ (p.395)  “We relied heavily upon the doctors to give us direction, and yet I felt like they did leave things up to us. They would try to point us in a general direction, but even then they would tell us “we can’t give you the answers, we can’t tell you what the right thing to do is.” It was kind of like presenting all of the options and possible outcomes.” (p. 395) |
| #10 | Tucker Edmonds et al. (2019) | Parent-informed physician-guided MDM | Tacit approval model | “Some participants did not recall being asked to make certain decisions, such as resuscitation of the infant, but recalled being asked to make others, such as route of delivery.  One woman explained: “At 24 weeks she told me that they do get very aggressive and the percentage of disability is like 20%, that is what she told me.”” (p. 822) |
|  |  | Physician-informed parent-guided MDM | Preference determination model | “However, at the same institution, another participant was allowed to make a decision regarding resuscitation of the infant before 24-week gestation. She said: “If you deliver early, um, they told me the options. Also, the option if I want to terminate the pregnancy knowing what I have to face. And the plan now, and they actually, I asked her to let me hold out as long as I could with the understanding that if I deliver in the 23rd (before 24 weeks) I would not resuscitate, that was my option.“” (p. 822) |
| #11 | Denney-Kölsch & Côté- Arsenault (2022) | Physician-informed parent-guided MDM | Preference determination model | “Parents carefully considered their options, needing time to process their concerns, and consulted extensively with their providers and each other. One mother with severe oligohydramnios shared that “We spent a significant portion of time talking about what would be the ramifications of each decision that we made…we wanted to know…from someone who wants as much information as possible, this is what I wanted to know but I didn’t know to ask.””(p. 5) |
| #12 | Orfali & Gordon (2004) | Parent-informed physician-guided MDM | Tacit approval model | “Physicians emphasized their expertise, providing parents with regular updates even when parents were too shy to come forward. A mother of a premature baby explained how she felt: “I had a total trust in the team…They were always straightforward, and nice and talking to you. You would ask questions and they would immediately answer. You had the impression that nothing was withheld from you, that everything concerning your child’s health was told. That was so important. To have a good doctor–parent relationship, a trust between us…well, as a physician I knew he would do the best for my child, I knew he would act ethically anyhow…” (p. 345) |
|  |  |  | Recommendation acceptance model | “Though parents were constantly asked to sign consent forms for  different medical procedures, they did not perceive themselves as  decision-makers. Actually, they (the doctors) make the decisions and we sign the consent forms…’’ (mother. Unit B) (p. 347) |
|  |  | Physician-informed parent-guided MDM | Preference determination model | “They were informed about their baby’s condition in either optimistic or highly uncertain terms: “The prognosis was very uncertain. Nobody knew what was going to happen’’ (Adriana, mother of a premature twin baby who stayed four months in the NICU).”  ‘Sometimes I was very afraid about what to do. (…) With the surgery, I didn’t know if it was good. His sister had the same one and she died in surgery’’ (Adriana). Still, Adriana did not want to give up her decision-making power: ‘‘No, I wasn’t asked to make too many critical decisions…’’ (p. 347) |
| #13 | Brinchmann et al. (2002) | Parent-informed physician-guided MDM | Tacit approval model | “The findings showed that most of them thought that, although parents should be well informed, consulted, listened to and included, it is the health professionals (doctors) who carry the responsibility (medical and legal), and should make the final decision.”(p. 394)  “It was an awful experience, that no one asked me what I wanted to happen. I remember asking the doctor whether we had any say, if they were going to ask us what we felt. ‘No’ they said, they never included the parents in these decisions; there was nothing to discuss.” (parent) (p. 394) |
|  |  |  | Recommendation acceptance model | “They stressed the value of seeming to be included, which means knowing that one does not decide, and that one is not given all the information, but that one does not feel that the decision is being taken over one’s head. This finding corresponds to Anspach’s ‘producing assent’ model: “I felt that we were seemingly being included, but that they wanted us to agree with them. I did not feel that the decision was being taken over our heads. It seemed to me as though they hoped that we would to some extent agree, I think so. So that we would not stand against them, that we were not completely opposed to them.”” (p.396) |
| #14 | Caeymaex et al. (2011) | Parent-informed physician-guided MDM | Tacit approval model | “Decision made by physician(s) No explicit parental involvement (tacit assent)” (p.4)  ‘‘As doctors, they considered that at some point it was necessary to decide to pull the plug.Therefore at that point, they suggested we all go to the bedside’’ (m38). (p.3) |
|  |  |  | Recommendation acceptance model | ‘The doctor said to me, ‘your opinion is of course important, and your decision will be equally important, but you should know that the medical team also has an opinion and a decision.’That was good. I said to myself, Thank god, it isn’t me who has to decide. Because I had just been thinking what a real, total fright it would be to decide alone’’ (m14) “ (p.3) |
|  |  | MDM with equal participation | Shared deliberation model | “Discussion on the nature of the decision Exchange of relevant medical information (medically reasonable alternatives) Exchange of family values and preferences Parental choice about most appropriate decision Consensus reached with physicians”  ‘‘The doctor left me the choice. He explained to me the risks of these choices. He told me, you already have a three-year-old daughter. He stayed in the context of our little family: for the child, for me, for my family. If something happens to you, who will take care of him? Very concrete questions’’ (p.4) |
|  |  | Physician-informed parent-guided MDM | Preference determination model | “Medical facts given by physician Deliberation and final decision by parents No discussion of values”  ‘‘He said to us that the team would follow us in our decision, that he thought it was a good decision. That was great solace, because in fact it was our decision to make, and it was horrible to decide. At the time, it was great to be able to decide, that is, if a doctor had said to me, ‘‘We are deciding this’’, it would have been unbearable for me. Here, it was difficult but the fact of having support, and hearing that … yes it did me a lot of good’’ (p.4) |
| #15 | Hendriks & Abraham (2017) | Parent-informed physician-guided MDM | Tacit approval model | “Participants whose infants died in the delivery room also wanted everything possible to be done to enhance the potential for survival; however, for some infants, care could not be initiated because of their prematurity or because of the hospital’s gestational cutoff policy.”  “We learned about this [the gestational age cut off] later. He was 23 weeks and some days. So that was the aggravating part of the story, so shortly before, why?. And that takes a while before you can accept it.” (p. 730-731) |
|  |  |  | Recommendation acceptance model | “What was really to decide? But actually. we relied on the staff and their advice. They have more experience, when they say it does not make any sense, then that was the decision. There was not really anything to decide.” (p. 731) |
|  |  | Physician-informed parent-guided MDM | Preference determination model | “Although decision making before birth is only possible when there is time, most participants were able to consider the option of steroids (for the maturation of fetal lungs) and/or make decisions regarding resuscitation or initiation of intensive care.”  “We had the option not to do anything and then she would die after a while. Or we had the option to try everything possible.. We discussed this and we said we would try everything.” (p. 731) |
| #16 | Geurtzen et al. (2019) | Parent-informed physician-guided MDM | Recommendation acceptance model | “That conversation was presented very positively – "we can do everything to save your child and that is definitely going to work and we have this percentage of being successful." And at some point that gave me the feeling that I couldn’t choose not to ripen the baby’s lungs. ( . . . ) They were so positive about the fact that they would do everything to support the baby that I almost felt like I didn’t have any other options, or well, that they would think I was really stupid . . . Or very cruel if I decided to go with the other option. And yes, that was the only thing that was missing for me.” (p. 1546) |
|  |  | Physician-informed parent-guided MDM | Preference determination model | “The good thing in our situation was that we had time between the conversations, that we did not have to make a decision during that conversation. We were then sort of able to think about things and make peace with what we had already decided during the first conversation, both of us I think. But it did give us time, to make that decision.” (p.1545) |
