# Supplementary material for: How parents derive their informed role preferences for end-of-life medical decision-making for (unborn) children in perinatology: a meta-ethnography of qualitative interview studies
Source: BMC Med Ethics. 2026 Sep 18;27:199. doi: 10.1186/s12910-026-01605-1 (PMC13589376; doi:10.1186/s12910-026-01605-1)
Supplement: Supplementary file 4 [file 12910_2026_1605_MOESM4_ESM.docx]

**Table S3 Change requests based on the evaluation of the experience**

| **No** | **Authors (year)** | **Passages from which a change request was derived** |
| --- | --- | --- |
| #1 | Wocial (2000) | “Feeling involved in discussions was central to parents' experience of considering withholding and/or withdrawing treatment from their infants. Feeling able to carry out their ethical obligations to their babies depended on parents' perceptions about being involved in discussions to consider withholding and/or withdrawing treatment from their babies. A negative example is a powerful demonstration of this theme. “They were supposed to call us and they never called. It was almost as if we had to grab them to find out what was going on. We would be visiting and someone would come to do a test and we had no idea what the test was for[horizontal ellipsis].” (p.83)  **—> preference for more guidance (being kept informed about the treatment plan)** |
| #2 | Kavanaugh et al. (2005) | “(...) all parents indicated that they had the right amount for their situation.” (...) “In contrast [to wanting to be involved], only one mother, who has a child who was born at 25 weeks’ gestation, felt that it was the physicians’ decision alone because of their expertise. She said, “They (physicians) want, they want to leave making my decision on what they do. But, I’m not a doctor, so it won’t be my decision. I’m trusting these guys with, with everything they do.” This mother’s infant died and at the postdeath interview, she indicated that the end-of-life decisions were also not hers to make. She felt that the physician should just initiate any treatment that is medically indicated. She said, “As long as the doctor is fully communicating, tell you exactly what’s going on, if, if this can work or if that can work and doctors know what can work and what cannot work, just tell us what you are going to do what’s going to work and just do it. . . . It wasn’t a decision for me to make (referring to withdrawal of ventilatory support). Whatever decision, everything they did for her, the doctors and the nurses made that decision. I didn’t make any decisions other than giving them confirmation over the phone for them to change the two lines and initially when she first got the blood transfusions.” (p. 354)  **—> preference for more guidance (leaving the decision to physicians and nurses)** |
| #3 | Payot et al. (2007) | **“**For parents, scientific information, although helpful to some extent, does not suffice on its own. On the one hand, the objective and neutral tone that accompanies the information they receive is experienced as detachment on the part of the clinicians.  **Parents 2 (2)**  “In fact, there isn’t really a real relationship. The relationship we had with them, it was one of authority. The specialist, he lays out his knowledge and then leaves.”  **Parents 1 (2)**  “The doctor comes, gives information and leaves, it’s like there is no relationship, someone you could rely on. If you are ill, you need to rely on someone and say ‘‘help me’’. There, you don’t want to say ‘‘help me’’ (y) he/she just comes, gives information and leaves.”” (...)  Detachment and lack of support from the medical team were thus repeatedly referred to by parents. (...)  **Parents 1 (2)**  “I think that what’s clearest now is really the lack of personal follow-up. No guidance. If there had been that, the rest would have been easier to deal with.”  **Parents 2 (1)**  “We don’t expect the physician to make the decision for us. But we need to feel that, well, that the physician... treats us more humanely... not like a client [to be able to deal with this intense situation].”  For parents then, a correct decision is one that takes into account their experience and that is supported by a scientifically competent and humane medical team. When looking back on their experience, these are the essential conditions that facilitate parents’ acceptance of this critical life event.  **Parents 2 (2)**  “It’s not something we want. It’s like, the final decision we make, it’s us that live with it, so we are the ones who are responsible. [y] so of course, we are the ones who live with the responsibility. So you have to be supported and guided.”” (p. 1494)  **—>preference for more guidance (shared deliberation with physicians)**  “On the other hand, the dark tone of the prenatal consultation, as well as the catastrophic vision that is portrayed by the bleak statistics, is interpreted as a lack of encouragement. Some parents felt that they had to justify their choice of giving the baby a chance.  **Parents 4 (2)**  “Are you sure, really sure that you want to keep her, and don’t imagine that it’s gonna be easy. It’s gonna be really hard, what you’re gonna have to go through.’’ It’s okay like that, that they say it like that, maybe. I don’t know... Shouldn’t they encourage new parents more?”  **Parents 5 (2)**  “I felt like I had a choice. Except that, I sorta had the feeling too that I had to give the reasons why I wanted... why it was my choice. In my opinion, I shouldn’t have to give a reason why I want that. It’s like if I had to argue that it’s my baby, that I want it, no matter what happens.”(p. 1494)  **—>preference for less guidance (no need to justify the preference)** |
| #4 | Boss et al. (2008) | - |
| #5 | Kavanaugh et al. (2009) | “When asked about shared decision making with the physician, John said it depends on how well you know, trust and respect the physician and their recommendations. He felt that the physician should give a recommendation and inform parents about all possible options, but that it should be up to the parents to make the final decision.” (p.6)  **—>preference for more guidance (physicians should give a recommendation)** |
| #6 | Moro et al. (2011) | - |
| #7 | Kavanaugh et al. (2015) | “Some parents felt that the providers had already decided on the treatment option and were just waiting for parents to agree with them. In these situations, parents described themselves as needing to remain strong to fight for decisions that they felt were in the best interests of their infant (Roscigno et. al., 2012).” (p.149)  **—> preference for less guidance (being able to determine the best interests of the child)**  “In contrast, when parents’ perspectives were not solicited, providers risked offering advice that at best was irrelevant, and at worst harmful: “The first day I went to [name of hospital], I came home crying, in tears, and [my partner] was like, “What’s wrong, what did they do to you?” And I told him, I said, “Well, they keep trying to get me to abort.” And he’s like, “What? What hospital is that? What, [do] they get a kickback from the abortion clinic or something?” And I said, “No, they just keep saying that everything will go wrong, everything will go wrong.” He was like, “What, I thought a hospital was to help sick people (. . .)” And so I knew from that point on, that’s their viewpoint and how they handle it. And it’s the one thing I do not like (. . .) because that makes me uncomfortable.” (p.149)  **—> preference for less guidance (not exerting pressure on the parent to implement an option)** |
| #8 | Daboval et al. (2016) | “The parents who were not satisfied with their participation felt that the physician had all the control over treatment decisions.  “I did not perceive that I had control of this, because I am not the physician, I am not the one performing the resuscitation, I feel that I don't have control of this...” (Mother-System #5)  If the physician had explored their wishes to participate (as was done in the PAC interview), he would have discovered that these parents felt that the decision should have been theirs and they should have been told that. They felt they did not have the opportunity to participate as they would have wished.”  “If the decision belongs to the parents, yes, in this case, yes [. . .]. It would have been nice to have been told that ‘in this case there are these risks and the decision is yours if you want to keep going or not’. . . “ (Mother-System #5) (...)  In one system (# 5), the father and the mother scores were 2 and 3 respectively, which we characterize as ªdissatisfiedº because of the great difference from the other systems and considering their PAC verbatim. Surprisingly, even though the parents rated the scores individually and silently; scores were similar between the two partners for all 5 systems. All physicians thought that parents were satisfied, giving perceived parent satisfaction scores between 7 and 10 including for the parents (system # 5) who were dissatisfied.” (p.6-7)  **—> preference for less guidance (being able to make the decision themselves)l** |
| #9 | Baughcum et al. (2017) | “Some parents cited that physicians were direct and upfront with them about their infant’s prognosis whereas other parents wished they would have been more forthcoming. (...)  “No one would volunteer their personal opinion. It felt strongly like everyone was recommending one thing but no one would outwardly say it. That made it tougher to know if we were doing the right thing (mother, 3 years after death).” (p. 397)  **—> preference for more guidance (physicians openly express their opinion on the options)** |
| #10 | Tucker Edmonds et al. (2019) | - |
| #11 | Denney-Kölsch & Côté- Arsenault (2022) | - |
| #12 | Orfali & Gordon (2004) | “The organizational experience and their personal needs did not match but seemed to produce constant ‘‘cognitive dissonance.’’The language of rights was not perceived at all in terms of empowerment. Parents in the American unit were provided with a lot of information without getting any specific emotional support to deal with it, in contrast with unit A.” (p.347)  **—>preference for more guidance (emotional support in dealing with the information)**  “In fact, many mothers are ambivalent about decision-making; on one hand they want to be involved and become angry if they are not given any control over many medical aspects of the baby’s care: ‘‘I wish that they had told me about the transfusion; no one called me…’’ On the other hand, no mother in our sample ever requested more active involvement in life and death decisions. They seemed to want some control over a dreadful situation, but none of them expressed eagerness to take over the terrible act of deciding life or death regarding their own child.” (p.347-348)  **—> preference for more guidance (being informed more often)** |
| #13 | Brinchmann et al. (2002) | “You have tried very hard and gone through a very intense mental process before you say, ‘Now I’ll let this little child of mine go, and that is pretty serious, don’t you think?’ While you are still in the process of having given birth you have to make an ethical decision about whether it is right to put in an enormous amount of resources and therapy just to see if it is at all possible to save this little life. As a mother I would rather be spared that. But I would really want to be well informed and participate in the process. I think that it is very important that we, as parents, are allowed to feel that we are participating, and that we get very clear feedback on whether we have been heard and which of the decisions are up to the doctors to decide.” (Interview 11 with parents; the infant survived without disability.) (p.396)  **—> preference for more guidance (being spared from decision-making, but being included and informed)**  “It is the parents who have to live with the consequences of these choices, whatever the result. Parents know themselves best and know best how they would be able to cope. It was further pointed out that adult patients have the right to decide for themselves, and that in other situations the opinions of close relatives are given weight when they are not capable of deciding for themselves. The question was raised concerning why neonatal medicine is different to other areas of medicine in this respect. One father, who was also a doctor, emphasized that parents’ observations and points of view must be taken seriously:  “We got to know our child very well. Even though this was their day-to-day work, their profession, none of the nurses came near to having half the observation time we [parents] had while we were there. There was no one who saw our child more than we did. That’s the way it is as a rule. It’s a golden rule they kept trying to knock into our heads in paediatrics at medical school, that we always have to listen to the child’s mother.” (Interview 5 with father/doctor; the infant died.)”” (p. 397)  **—> preference for less guidance (being able to decide for the child)** |
| #14 | Caeymaex et al. (2011) | “Several stressed the difficulty of having to decide alone. ‘‘The doctors say to us: ‘It’s your choice. We are leaving the decision to you’. And finally, that is very very hard. I don’t think it is a good thing’’ (f21).” (p.4)  **—>preference for more guidance (not being on their own)**  “A smaller number of parents (11 parents out of the 78) expressed guilt feelings directly related to their role in the EOL DMP. Amongst these 11 parents, 3 perceived an informed parental decision and the remaining 8 a shared decision. A mother who perceived she had decided without the doctors, reported: ‘‘We are the ones who said, then, on such a day, we stop. It is difficult for parents to tell themselves that they are not (well let’s say) ‘‘killing’’ our child; it is that you stop, we stopped what was keeping her alive. You hate yourself’’ (m27).”  (p.5)  **—>preference for more guidance (not carrying the responsibility alone)**  „Parental desire for guidance in the DM varied amongst participants. More than half of the participants stated that the medical staff should express their opinions overtly and directively. These parents reported that they had felt overwhelmed by the situation (emergency, discovery of an unexpected malformation, or extreme prematurity) or by the exhaustion due to the baby’s long hospital stay. Some mothers related this to their own weak health status in the post-partum. (p.5)  **—>preference for more guidance (physician should express their opinions overtly and directively)**  “Other parents (approximately a quarter of the participants) preferred that the staff reveal its preference non-directively. Finally, a small minority reported that they did not need the staff opinion to decide.” (p.5)  **—>preference for less guidance (physicians should communicate the options non-directively)** |
| #15 | Hendriks & Abraham (2017) | “Participants perceived unsatisfactory decision making because of communication challenges. At the prenatal stage, participants reported that they were not always informed about the hospital’s gestational cutoff policy, that is, the non-initiation of treatment for infants born before 24 weeks gestation. Some participants received only implicit or minimal information on the hospital’s guidelines, which prompted them to actively seek full and explicit information. Other participants did not receive any type of prenatal information, and no transparent reasons for why treatment was withheld at the prenatal stage were given: “We learned about this [the gestational age cut off] later. He was 23 weeks and some days. So that was the aggravating part of the story, so shortly before, why?. And that takes a while before you can accept it.” (Mother)” (p. 730-731)  **—>preference for less guidance (no strict hospital policy)**  However, some participants were dissatisfied with their level of involvement and felt that the options and decisions presented by the health care team might have been biased and directive:  “The doctors make strong suggestions so that you cannot really decide ... you do what the hospital offers you. A different hospital might act differently ... possibly there were other options, but when you do not know what other options there are, how should you decide? (Father)“ (p. 732)  **—>preference for less guidance (less biased and directive communication)** |
| #16 | Geurtzen et al. (2019) | “That conversation was presented very positively – "we can do everything to save your child and that is definitely going to work and we have this percentage of being successful." And at some point that gave me the feeling that I couldn’t choose not to ripen the baby’s lungs. ( . . . ) They were so positive about the fact that they would do everything to support the baby that I almost felt like I didn’t have any other options, or well, that they would think I was really stupid . . . Or very cruel if I decided to go with the other option. And yes, that was the only thing that was missing for me.” (Table 3B, p.1546)  **—>preference for less guidance (being offered multiple options)** |
